# Sexual conflict and consistency of offspring desertion in Eurasian penduline tit *Remiz pendulinus*

**DOI:** 10.1186/1471-2148-8-242

**Published:** 2008-09-01

**Authors:** Ákos Pogány, István Szentirmai, Jan Komdeur, Tamás Székely

**Affiliations:** 1 Department of Ethology, Eötvös University, Pázmány Péter S. 1/C, Budapest, H-1117, Hungary; 2 Animal Ecology Group, Centre for Ecological and Evolutionary Studies, University of Groningen, Biological Centre, P.O. Box 14, 9750, AA Haren, The Netherlands; 3 Department of Biology and Biochemistry, University of Bath, Bath, BA2 7AY, UK

## Abstract

**Background:**

The trade-off between current and future parental investment is often different between males and females. This difference may lead to sexual conflict between parents over care provisioning in animals that breed with multiple mates. One of the most obvious manifestations of sexual conflict over care is offspring desertion whereby one parent deserts the young to increase its reproductive success at the expense of its mate. Offspring desertion is a wide-spread behavior, and its frequency often varies within populations. We studied the consistency of offspring desertion in a small passerine bird, the Eurasian penduline tit *Remiz pendulinus*, that has an extremely variable breeding system. Both males and females are sequentially polygamous, and a single parent (either the male or the female) incubates the eggs and rears the young. About 28–40% of offspring are abandoned by both parents, and these offspring perish. Here we investigate whether the variation in offspring desertion in a population emerges either by each individual behaving consistently between different broods, or it is driven by the environment.

**Results:**

Using a three-year dataset from Southern Hungary we show that offspring desertion by females is consistent between nests. Male desertion, however, depends on ambient environment, because all males desert their nests early in the season and some of them care late in the season. Therefore, within-population variation in parental care emerges by sexually different mechanisms; between-individual variation was responsible for the observed pattern of offspring desertion in females, whereas within-individual variation was responsible for the observed pattern in males.

**Conclusion:**

To our knowledge, our study is the first that investigates repeatability of offspring desertion behavior in nature. The contrasting strategies of the sexes imply complex evolutionary trajectories in breeding behavior of penduline tits. Our results raise an intriguing question whether the sexual difference in caring/deserting decisions explain the extreme intensity of sexual conflict in penduline tits that produces a high frequency of biparentally deserted (and thus wasted) offspring.

## Background

Evolutionary interests of males and females are often different over reproduction (sexual conflict; [1]). Such difference may emerge from divergent optima over the number of matings [2-5], or over provisioning the offspring by the parents [6,7]. Since the benefit of rearing young is shared approximately equally by the biological parents (but make allowances for genomic imprinting [8,9]), whereas each parent pays the cost of caring itself, the best interest of parents is often to shunt care provisioning to their mate [6,7]. One of the most obvious manifestations of sexual conflict between parents is offspring desertion whereby one parent leaves the burden of care provisioning to its mate [10].

Offspring desertion occurs in a variety of organisms including insects, fishes, amphibians, birds and mammals [11-13]. Typically one sex abandons the young, for instance, in mammals it is usually the male that withholds care, whereas in majority of fishes the female does so [13]. Desertion is beneficial for the deserting parent, since it improves his/her chances for reproduction in future, whereas it is costly for the abandoned mate in terms of time and energy spent on reproduction [7,14-17]. In a handful of species, however, either the male or the female may abandon the young, and leave the provisioning of full care to its mate [10,12]. In these species, behavior of an individual may depend on the behavior of its mate as well as behavior of other individuals in the population [12,18-21]. Therefore, full understanding of care and desertion patterns requires a game-theoretical analysis that includes (but not restricted to) costs and benefits and the process of interactions [22,23].

In any given population, variation in parental care behavior may emerge in three ways. First, individuals may have different propensities to desert or care, and this propensity is consistent for a given individual over a breeding season, or over its lifetime. Second, each individual exhibits variable behavior, and this variation is driven by environmental cues, such as differences in day length (i.e. time in the season), habitat quality, or operational sex ratio (the ratio of sexually receptive females and males, e.g. [24]). Third, each individual behaves randomly. Although understanding parental decisions is fundamental for predicting breeding systems and the evolution of sex roles [18,25-28], it is striking that no study has yet tested the consistency of caring/deserting decisions in a natural population.

We investigated the repeatability in caring/deserting behavior in a species with unusually variable breeding system, the Eurasian penduline tit *Remiz pendulinus*. In this small passerine bird (body mass is about 9 g) both sexes are sequentially polygamous, and either the male or the female may desert the clutch and leave the full task of incubation and brood-rearing to its mate during egg-laying, before incubation starts (table 1, [29]). The deserting parent often re-mates shortly after abandoning the nest, so that both males and females may have up to seven mates in a single breeding season [29,30]. A striking feature of penduline tits' breeding system is the high frequency of biparentally deserted clutches (28–40%, table 1). These biparentally deserted (and thus failed) clutches appear to be the outcome of intense sexual conflict [2,30], whereby each sex attempts to increase its own reproductive success even if this is costly to its mate. Consistent with this suggestion is that desertion is beneficial for the deserting individual, although costly to its mate [30]. For instance, desertion by the male increases his own, but reduces his mates' total number of nestlings in the season. Interestingly, the sexually antagonistic interests are mirrored by the sexes, so that desertion by the female is beneficial for herself, but costly to her mate [30].

**Table 1:** Frequencies of parental care in four European populations of penduline tit *Remiz pendulinus*.

Population	Female-only care (%)	Male-only care (%)	Biparental desertion (%)	Reference
Sweden (*N *= 140 nests)	48	18	34	[29]
Germany (*N *= 89 nests)	65	7	28	[60]
Austria (*N *= 107 nests)	54	14	32	[60]
Hungary (*N *= 291 nests)	49	11	40	[61]

In all populations a single penduline tit (either the male or the female) cares for the eggs and chicks. Biparental care is extremely rare [59], and it was not found in any of these studies. Note the high frequency of biparental desertion in all populations.

Here we use a three-year data set of Eurasian penduline tits in Southern Hungary to investigate the repeatability of caring/deserting behavior in two contexts. First, we investigate whether desertions by males and females are consistent between subsequent nests (consistency analysis, henceforward). We prefer the term 'consistency' over 'repeatability', because in repeatability analysis the traits typically have normal distribution, so that General Linear Models (GLMs) can be used to separate within- and between-individual variations [31,32]. Caring/deserting, however, is a binomially distributed trait and we used Monte Carlo Simulation [33]. Second, we tested whether ambient environment influenced caring/deserting behavior. Specifically, we tested whether individual behavior changes over the breeding season (trend analysis, henceforward). Since both abiotic and biotic variables (e.g. ambient temperature, day length, food availability) and the social environment (e.g. the number of potential mates) often vary over the breeding season, desertion behavior, if it depends on some of these variables, should reflect seasonal variation.

## Results

### Consistency of parental care

Caring/deserting behavior of males was over-randomized (*P *= 0.991, Δ*C*_*crit *_= 0.801, *N *= 57 males); thus if a male deserted one of his nests, he was more likely to care for his next nest. Female behavior, however, was consistent between nests (*P *= 0.037, Δ*C*_*crit *_= 0.650, *N *= 20 females).

### Seasonal trend in parental care

**Figure 1:**
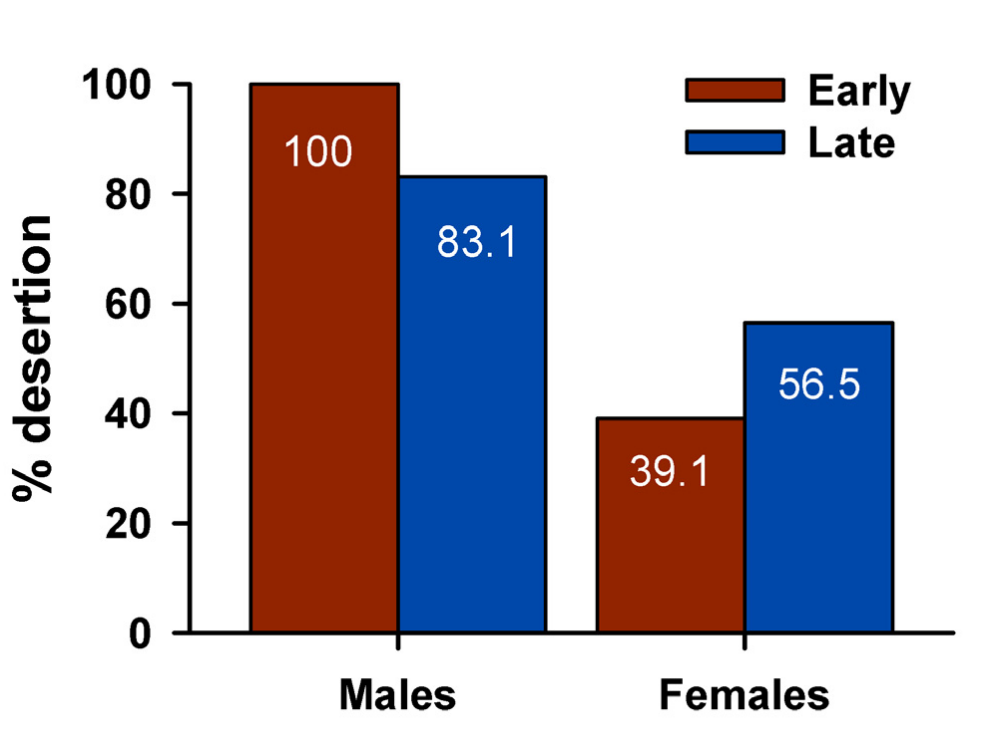
**Nest desertion by male and female Eurasian penduline tits**. Males desert their nests early in the season, and some of them care for their late ones (*P *< 0.001, *χ*^2 ^= 13.075, *N *= 146 nests). Female behavior is not different between early and late nests (*P *= 0.767, *χ*^2 ^= 0.088, *N *= 46 nests).

Concordantly with the results of consistency analysis (see above), males changed their behavior with advance of the breeding season. Males uniformly deserted early in the season although some males cared later in the season (*P *< 0.0001, Δ*T*_*crit *_= 0.199, *N *= 57 males; figure 1). Female behavior, however, did not change over the breeding season (*P *= 0.148, Δ*T*_*crit *_= -0.150, *N *= 20 females; figure 1).

## Discussion

We revealed sexual differences between parental care decisions of male and female penduline tits. Our findings are in line with recent studies of repeatability and genetic differences of parental behaviors [34-38], and a further step to understand parental care decisions and the evolution of breeding systems in nature.

Our main results are that female penduline tits are consistent in their desertion behavior, and male behavior is predicted by ambient environment, in terms of early *versus *late season. Female behavior varied little between subsequent nests, and they either cared or deserted consistently regardless of time in the season. We propose three explanations for this pattern. First, female penduline tits may vary in some traits linked to mating success, and this, in turn, would affect their care decisions. For instance, attractive (or fecund) females may desert more frequently than non-attractive (or less fecund) ones, since they are likely to re-mate sooner. To investigate this proposition, further work focusing on female attractiveness, fecundity and male preference is needed. Second, energy demands of various stages of reproduction may be different, and this predicts state-dependent parental decisions [21,39]. In line with the latter suggestion, weather conditions have frequently been reported to predict offspring desertion in various species [40-42], besides, in a recent study Bleeker et al. [43] found that offspring desertion is influenced by body condition in penduline tits. Therefore, it is possible that body condition of female penduline tits changes slower than that of males, and this results in consistent parental behavior in females while not in males. Third, consistent parental decisions of females may be the result of fixed genetic effects and/or imprinting in parental behavior [35,37,39]. For instance, by crossing two types of sticklebacks *Gasterosteus aculeatus *with a different propensity to care, Blouw [44] demonstrated that parental behavior is heritable in laboratory circumstances. Testing heritability of caring/deserting in penduline tits, however, is challenging in nature, because offspring recruitment is low (6.0% for males, 7.2% for females, van Dijk et al. unpublished data).

In contrast to females, parental behavior of male penduline tits depends upon the timing in the season. We suggest this seasonal trend reflects changes in circulating hormonal levels, or seasonal variation in the sensitivity of the receptors of breeding-related hormones [45]. Studies with passerine birds show that individuals breeding early in the season have higher testosterone levels than those breeding later [45,46]. Testosterone level is a key component in the trade-off between male mating effort and parental care, because high testosterone levels stimulate sexual behavior (such as male-male competition or nest guarding), whereas it suppresses paternal care [45-49]. Testosterone also plays a role in the development of ornaments [50,51]. Consistently with these studies, male penduline tits (but not females) molt late in the season [SA Kingma, personal observation] when their testosterone level is presumably low [46]. Therefore, timing of molting corresponds to male care, thus seasonal change in testosterone levels seems a promising candidate for explaining the change in male parental care [52].

Different physiology of male and female penduline tits may contribute to the different individual strategies we showed here. Female penduline tits continue producing eggs throughout the breeding season which is unusually long, approximately 3.5 months in Hungary. If sexual hormones (e.g. prolactin) are associated with egg-laying, then these may maintain consistent behavior throughout the season. Males, however, may have high testosterone levels early in the season that helps them to acquire mates, and as the breeding season progresses, their testosterone level may gradually decline. Coupled this with the declining number of females that are available (since most females are tied up with caring in the population), the propensity of males may change from desertion to provide care. In order to test these propositions, we need to investigate the physiological mechanisms responsible for desertion, and/or manipulate circulating hormone levels.

How are the different strategies maintained in a population? We propose two explanations for the existence of different male and female strategies. First, the seasonal trend in males, and the consistent behavior in females may be an optimal pair of strategies. For a male, deserting early in the season is beneficial, since if his female is a 'caring' type his offspring will be catered for, whereas if his female is a 'deserting' type she may carry his sperm and fertilize eggs in her new clutch. Late in the season, however, both of these benefits of desertion diminish for the male. Currently we are testing this proposition by genotyping chicks and adults (Mészáros et al. in prep). From the female perspective, deserting early in the season looks like a costly strategy that may be balanced out by the benefit of deserting late in the season – when males are more likely to care. For females of the caring type, these costs and benefits may be reversed over the breeding season: they benefit early in the season but pay a cost later. Whilst these arguments have their intuitive appeal, a proper understanding of the penduline tit breeding system requires a full game-theoretic model (van Dijk et al. in prep).

Second, the observed strategies may not be optimal, and the low breeding success reduces population viability. Fully fixed behavioral strategies (care/desert) would not be stable in the population, because the other sex was to exploit the fixed strategy due to sexual conflict. The high frequency of biparentally deserted nests in different European populations, however, suggests that the reproductive success of these populations is not at the maximum (table 1). Biparental desertions can be viewed as 'mistakes', since each sex assumes the other sex will care for the clutch, whereas in reality it may have already deserted. We are currently pursuing the latter proposition by analyzing the behavior of males and females immediately preceding desertion (van Dijk et al, in prep). Biparental desertion occurs during egg-laying, and it implies that the male wastes his energy and time (often, weeks) building a sophisticated nest, and then the female wastes her effort on producing the clutch of up to 5 eggs. Our analyses suggest that the high frequency of biparental desertion emerge when the population consists of many females from the 'deserting' phenotype, and it is early in the season so that the males also desert. Further studies by monitoring penduline tits' population dynamics may reveal whether immigration/emigration of females with different tactics contribute to the observed patterns of offspring desertion.

Our results contribute to the different repeatabilities of male and female parental behavior reported in other studies. Potti et al. [34] showed that female pied flycatchers *Ficedula hypoleuca *spend repeatable amount of energy on parental care between breeding seasons, whereas the energy expenditure of males was not repeatable. Schwagmeyer and Mock [36] and Nakagawa et al. [53] reported food provisioning levels to be repeatable in male house sparrows *Passer domesticus*, but not in females. However, MacColl and Hatchwell [54] found both male and female feeding rates of long-tailed tits *Aegithalos caudatus *were repeatable. In addition, a recent study by Charmantier et al. [55] showed high heritability in cooperative behavior in male Western bluebirds *Sialia mexicana*. These studies together with our findings suggest that individuals of one sex may be more variable in their parental care, thus sexual differences may emerge over the repeatability/flexibility of parental care.

Establishing the repeatability (or heritability) of behavior does not negate the influences of environment on parental behavior. For instance, food availabilities, predation, and operational sex ratio may all be involved influencing care provisioning (reviewed by [7,56,57]). In addition to these ecological traits the behavioral interactions may also influence conflict resolution. Recently we showed that at biparentally deserted nests the male and female desert on the same day [23]. The latter result raises the intriguing possibility that desertions may not be independent by males and females [12]. To explore this proposition, one needs larger sample sizes for powerful statistical analyses that can distinguish between competing theoretical scenarios. We suspect that ecological variables and genetic (or learnt) predispositions may interact, and this further underlies the significance of larger datasets than those we currently have, and the need of experimental manipulations.

## Conclusion

We analyzed within-population variation in offspring desertion in a small passerine bird that exhibits one of the most complex parental care systems in birds. We showed that female penduline tits have consistent parental decisions regardless of time in the breeding season, whereas male behavior is largely driven by timing in the season. Therefore, within-population variation in parental care emerges differently for males and females, since variation in female behavior at population level mainly emerges by between-individual, whereas variation in male behavior is mainly due to within-individual variation. These contrasting strategies suggest complex evolutionary trajectories in breeding behavior of species with variable breeding system.

## Methods

### Study site and data collection

Data were collected at Fehértó (46°19'N, 20°5'E), an extensive fishpond system in Southern Hungary, between 1 April and 19 August each year (2002 – 2004) that included the main breeding season. Penduline tits build nests on trees (largely, poplar *Populus spp. *and willow *Salix spp.*) along the dykes separating the fishponds. Nest-building males were searched on most days during the breeding season, and males were mist-netted when building their first nest using song playback and a male penduline tit dummy [43,58]. Female penduline tits were caught either together with their mate during mist-netting, or they were caught in the nest during incubation. Penduline tits were banded by a metal band of the Hungarian Ornithological Institute, and three color rings (A. C. Hughes, Middlesex, UK) that allowed us to identify the individuals from a distance using binoculars. Nests of mated pairs were checked approximately daily. Desertion by the male and/or the female was established if the given individual was not observed at the nest for 30 min on two consecutive days [23].

### Data processing

For each individual all nests in a given year were included in the analyses. If an individual had multiple nests from more than one year (3 out of 60 males, 1 out of 21 females), either the year with the highest number of nests was included, or in case of equal number of nests we chose a year randomly. We constructed one data set each for males and females. The same data sets were used for the analyses of both consistency and seasonal trend. In each data set, rows represented individuals and columns represented their subsequent nests. Score 1 and 0 indicated nest desertion and care, respectively.

In both male and female data sets, only individually banded males and females were included, respectively. Female data set has smaller sample size, since females are more difficult to trap. The number of nests and the proportion of desertions are given in table 2. These sample sizes are larger than those in former studies of caring/deserting behavior (see McNamara et al. [12]).

**Table 2:** Summary of nests used in randomizations.

	MALE	FEMALE
No. of individuals	57	20
No. of individuals per year	19 ± 2.6	6.7 ± 1.8
No. of nests	157	53
No. of nests per year	52.3 ± 8.5	17.7 ± 5.2
No. of nests per individual	2.75 ± 0.15	2.65 ± 0.17
Deserted nests (%)	91.7	49.1
Deserted nests per year (%)	91.3 ± 2.1	50.4 ± 12.7
Desertion date (no. of nests)		
Early nests	67.9 ± 2.1 (72)	51.7 ± 5.1 (22)
Late nests	94.1 ± 1.4 (72)	87.4 ± 3.1 (23)
Mann-Whitney *U*	411	65.5
*P*	< 0.0001	< 0.0001

Number of nests used in randomizations for males and females (mean ± SE). Two data sets were constructed in which male and female penduline tits were analyzed separately. Desertion date of nests is the number of days since 1 April in each year. *U *and probability (*P*) of Mann-Whitney tests (early *versus *late nests for each data set) are also provided.

### Desertion consistency analysis

For each individual we calculated the absolute differences between his/her scores for all possible comparisons between two nests. For example, if an individual had three nests (a, b, c), the differences between scores of all possible nest pairs were calculated as |a - b|, |a - c|, and |b - c|. Then for each individual the proportion (*p*) of consistent decisions between nest pairs was calculated as

(1) *p *= no. of nest pairs where difference is zero/no. of all possible comparisons

The mean of these proportions across individuals was taken as the critical value of test statistic (Δ*C*_*crit*_).

Then each observation was randomly allocated into a position without replacement, thus randomization preserved all observations and the data structure. Randomization was iterated 10^4 ^times, and at each iteration the test statistic (Δ*C*) was calculated as above. Randomization was carried out by Resampling Stats for Excel (2006). Finally, the probability of Δ*C *larger than Δ*C*_*crit *_was calculated (*P*), and we report this value.

### Trend analysis

Each row in the data sets was divided into first and second half, representing nests built during early or late breeding season, respectively. Rows with an odd number of nests had the middle nest eliminated. Early versus late nests for a given individual correspond to early and late calendar dates of nest desertion (see table 2). Relative desertion dates (number of days from 1^st ^of April in each year) of early *versus *late nests differed in both male and female data sets (table 2).

The mean score of early nests and late nests was calculated separately; for instance, an individual with desertion history 1,1,0,1, the means of early and late nests were 1 and 0.5, respectively, whereas for an individual with desertion history 1,0,1,1,0, the corresponding means were 0.5 and 0.5. Then the mean score of late nests was subtracted from the mean score of early ones, and finally, the test statistic (Δ*T*_*crit*_) was calculated as the mean of all these differences. For the two individuals in the preceding example Δ*T*_*crit *_= (0.5 + 0)/2 = 0.25. Therefore, positive Δ*T*_*crit *_indicates more desertion early in the season than later, whereas a negative Δ*T*_*crit *_indicates *vice versa*. Accordingly, values close to zero indicate no seasonal change in care pattern.

In trend analyses the randomization followed the same logic as in consistency analysis (see above), so that the mean difference (Δ*T*) was calculated in 10^4 ^iterations. We then took the probability (*P*) of higher (if Δ*T*_*crit *_was positive), or lower (if Δ*T*_*crit *_was negative) Δ*T *than the test statistic.

## Authors' contributions

AP performed statistical analysis and drafted the manuscript in partial fulfillment of a doctoral degree at Eötvös University. IS was involved in acquisition of data, coordination of fieldwork and revision of the manuscript. JK assisted with editing and revision of the manuscript. TS conceived of the study, contributed to data, and assisted in the design of the study, editing and revision of the manuscript. All authors read and approved the final manuscript.
